# Occurrence, antimicrobial susceptibility, and pathogenic factors of *Pseudomonas aeruginosa* in canine clinical samples

**DOI:** 10.14202/vetworld.2021.978-985

**Published:** 2021-04-23

**Authors:** Jasmine Hattab, Francesco Mosca, Cristina Esmeralda Di Francesco, Giovanni Aste, Giuseppe Marruchella, Pierluigi Guardiani, Pietro Giorgio Tiscar

**Affiliations:** 1 Department of Veterinary Medicine, University of Teramo, Loc. Piano d’Accio, 64100, Teramo, Italy; 2 Veterinary Clinic “Saline”, Città Sant’Angelo, 65013, Pescara, Italy

**Keywords:** antimicrobial susceptibility, biofilm, dog infection, extracellular pathogenic factors, *Pseudomonas aeruginosa*

## Abstract

**Background and Aim:**

*Pseudomonas aeruginosa* is a relevant opportunistic and difficult to treat pathogen due to its widespread environmental diffusion, intrinsic resistance to many classes of antimicrobials, high ability to acquire additional resistance mechanisms, and wide range of pathogenic factors. The present study aimed to investigate the prevalence of *P. aeruginosa* in canine clinical samples, the antimicrobial susceptibility against antipseudomonal antibiotics, and the presence of extracellular pathogenic factors of the isolates, as well as their ability to produce biofilm.

**Materials and Methods:**

Overall, 300 clinical specimens from dogs with pyoderma or abscesses (n=58), otitis (n=59), and suspected bladder infection (n=183) were analyzed by standard bacteriological methods. *P. aeruginosa* isolates were tested for their antimicrobial susceptibility by disk and gradient diffusion methods to determine the minimum inhibitory concentrations. The ability of the isolates to produce biofilm was investigated by a microtiter plate assay, while virulence genes coding for elastase (*lasB*), exotoxin A (*toxA*), alkaline protease (*aprA*), hemolytic phospholipase C (*plcH*), and exoenzyme S (*ExoS*) were detected by polymerase chain reaction method.

**Results:**

A total of 24 isolates of *P. aeruginosa* were found in clinical specimens (urine n=3, skin/soft tissue n=6, and ear canal n=15). No resistance was found to ceftazidime, gentamicin, aztreonam, and imipenem (IMI), while low levels of resistance were found to enrofloxacin (ENR) (4.2%) and piperacillin-tazobactam (8.3%). However, 41.7% and 29.2% of the isolates showed intermediate susceptibility to ENR and IMI, respectively. Disk and gradient diffusion methods showed high concordance. The majority of the isolates revealed a weak (33.3%) or intermediate (45.8%) ability to form biofilm, while the strong biofilm producers (20.8%) derived exclusively from the ear canal samples. All isolates (100%) were positive for *lasB*, *aprA*, and *plcH* genes, while *exoS* and *toxA* were amplified in 21 (87.5%) and 22 (91.7%) isolates, respectively.

**Conclusion:**

In the present study, *P. aeruginosa* isolates from canine clinical samples were characterized by low levels of antimicrobial resistance against antipseudomonal drugs. However, the high presence of isolates with intermediate susceptibility for some categories of antibiotics, including carbapenems which are not authorized for veterinary use, could represent an early warning signal. Moreover, the presence of isolates with strong ability to produce biofilm represents a challenge for the interpretation of the antimicrobial susceptibility profile. In addition, the high prevalence of the extracellular pathogenic factors was indicative of the potential virulence of the isolates.

## Introduction

*Pseudomonas aeruginosa* is a Gram-negative, rod-shaped, non-glucose-fermenting aerobic bacterium that is widely distributed in environment [1]. It represents an opportunistic pathogen in dogs, mainly involved in ear canal and soft-tissues infections [2] and less commonly in urinary tract infection [3]. *P. aeruginosa* is naturally resistant to many classes of antimicrobial drugs due to intrinsic mechanisms, such as low outer membrane permeability [4], constitutive efflux pump systems [5], and high basal expression of chromosomal AmpC β-lactamase [6]. Hence, therapeutic options for *P. aeruginosa* are limited and only a few antibiotics are useful as antipseudomonal drugs, most of them not licensed for veterinary use. In addition, *P. aeruginosa* can rapidly acquire resistance through overexpression of efflux pumps [7], loss of functionality of porins [8], mutational changes of the antibiotic targets [9], and expression of a wide range of β-lactamases [10]. Therefore, *P. aeruginosa* can achieve resistance to each antimicrobial class through single or multiple mechanisms [11].

The worldwide emerging of resistant strains represents a serious threat for both human and animal health, considering the human-pet relationship as a critical issue for the transmission of clinically significant multidrug-resistant bacteria [12].

The present study aimed to investigate the prevalence of *P. aeruginosa* in canine clinical samples, the antimicrobial profile, and the presence of extracellular pathogenic factors of the isolates, as well as their ability to produce biofilm.

## Materials and Methods

### Ethical approval

Ethical approval was not needed since the present study was performed on diagnostic samples collected during the routine veterinary activities and submitted to the bacteriological investigation for diagnostic purpose.

### Samples, study period, and bacteriological investigation

Overall, 300 clinical specimens from dogs with pyoderma or abscesses (n=58), otitis (n=59), and suspected bladder infection (n=183) were analyzed from May 2019 to July 2020. The specimens were non-duplicate and they were presumed to be epidemiologically non-related since they came from different animals, different families, presented different sampling dates, and originated from different locations. Samples were cultivated aerobically on 5% sheep blood agar and MacConkey agar at 36°C for 24-48 h. A sample was considered positive if the bacterial growth was significant, both as pure culture or mixed microbial flora with dominance of one bacterial species. Subcultures of the positive samples were obtained using tryptone soya agar, then bacteria were Gram-stained and tested for catalase and oxidase. Confirmation of *P. aeruginosa* isolates was performed using a commercial biochemical identification system (API^®^ 20 NE, BioMerieux, France).

### Antimicrobial susceptibility testing (AST) of P. aeruginosa isolates

Colonies of *P. aeruginosa* were suspended in normal sterile saline solution at optical density (OD) of 0.1, measured through spectrophotometer at 595 nm. The suspensions were swabbed on Mueller-Hinton agar (MHA). AST was performed by gradient diffusion method (Mic Test Strip™, Liofilchem, Italy), and after incubation at 36°C for 18 h, the minimum inhibitory concentrations (MICs) of gentamicin (CN), enrofloxacin (ENR), ceftazidime (CAZ), imipenem (IMI), aztreonam (ATM), and piperacillin-tazobactam (TZP) were determined for each isolate. The antimicrobial concentration range was 0.016-256 μg/mL for CN, ATM, CAZ, and TZP and 0.002-32 μg/mL for ENR and IMI. Specific Clinical and Laboratory Standards Institute interpretative criteria for *P. aeruginosa* were considered to categorize each isolate as susceptible, intermediate, or resistant. In particular, human breakpoints were adopted for CAZ, IMI, and ATM [13], and veterinary breakpoints for CN, ENR, and TZP [14]. For each antibiotic, MIC_90_ and MIC_50_ values were defined as the lowest concentration at which 90 and 50% of the isolates were inhibited, respectively. The results obtained from the gradient diffusion method were compared to the results of the disk diffusion method that was performed using the same bacterial inoculum [15]. Inhibition zone diameters on MHA were measured after incubation at 36°C for 18 h and the categories were elaborated according to human breakpoints for CAZ (30 μg), IMI (10 μg), and ATM (30 μg) [13] and to veterinary breakpoints for CN (10 mg) and ENR (5 mg) [14]. *P. aeruginosa* ATCC^®^ (USA) 27853 was used for quality control test. Considering the disk diffusion method as the reference standard, we classified the discrepancies between both methods into three categories, corresponding to very major (false-susceptible gradient diffusion test result), major (false-resistance gradient diffusion test result), and minor (difference in one interpretation category) [16].

### Phenotype screening of β-lactamase production

The isolates with intermediate/resistant profile to one or more b-lactams were tested by combination disk tests for the detection of AmpC β-lactamase, extended-spectrum β-lactamases (ESBLs), and metallo-β-lactamases (MBLs). Bacterial isolates were suspended in normal sterile saline solution at 0.1 OD_595_ and then swabbed on MHA.

### Phenotype screening of AmpC overproduction

According to the method commonly used in *Enterobacteriaceae* [17], disks containing CAZ (CAZ 30 μg) and cefotaxime (CTX 30 μg) alone and in combination with cloxacillin were placed on MHA. The test was considered positive if the inhibition zone diameters of both cephalosporins plus cloxacillin were ≥5 mm larger than cephalosporins alone.

### Phenotype screening of ESBLs

A previously described method for *Enterobacteriaceae* was adopted [13] with some modifications [18]. Disks containing CAZ (CAZ 30 μg) and cefotaxime (CTX 30 μg) alone and in combination with clavulanic acid (10 μg) were placed on MHA containing cloxacillin at 250 μg/mL. The test was considered positive if the inhibition zone diameters of both cephalosporins plus clavulanic acid were ≥5 mm larger than cephalosporins alone.

### Phenotype screening of MBLs

As previously described [19], disks containing IMI (10 μg) alone and in combination with EDTA (750 μg) were placed on MHA containing cloxacillin at 250 μg/mL. The test was considered positive if the inhibition zone diameter of IMI plus EDTA was ≥7 mm larger than IMI alone.

### Biofilm production

Biofilm production was assessed by microtiter plate assay, as previously described [20,21]. *P. aeruginosa* cultures were inoculated into Luria Bertani (LB) broth to create a suspension with OD_595_ of 0.1 and 200 μL of each isolate were then placed in triplicate into 96-well polystyrene microtiter plate. After incubation for 48 h at 36°C under static condition, the supernatants of the wells were transferred to empty wells and their OD_595_ values were measured. Then, the wells containing potential biofilm were kindly washed 3 times and the plate was dried at 60°C for 1 h. Biofilms were stained with 200 μL of a crystal violet solution (0.1% w/v) for 15 min at room temperature (25ºC) and the wells were washed 3 times to remove the excess stain. The microplate was air-dried, and for each well, the stained biofilms were solubilized with 200 μL ethanol (95%). After 15 min at room temperature (25ºC), the OD values of each well were measured at 570 nm. The wells containing non-inoculated LB broth represented the negative control and the relative OD values were subtracted to the OD values of the wells containing bacterial samples. Biofilm index (BI) of each isolate was calculated as the OD_570_/_595_ ratio, reflecting the proportion between biofilm production and planktonic growth. Based on the mean BI values obtained from four replicate experiments, each isolate was classified into three categories, corresponding to strong (≥2), intermediate (1-2), and weak biofilm producer (≤1) [22].

### DNA extraction and pathogenic factors detection

DNA extraction was performed by QIAamp DNA Mini Kit (Qiagen, Germany) according to the manufacturer’s instructions. Virulence genes coding for elastase (*lasB*), exotoxin A (*toxA*), alkaline protease (*aprA*), hemolytic phospholipase C (*plcH*), and exoenzyme S (*ExoS*) were detected by polymerase chain reaction (PCR) method, as previously reported [23,24] (Table-1).

**Table 1:** Primers and protocols used for PCR detection of the virulence genes.

Gene target	Sequences (5’- 3’)	Amplicon size (bp)	Denaturation	Amplification	Final extension	Ref
lasB-F	GGAATGAACGAAGCGTTCTC	300	94°C – 3 min	30 cycles	72°C – 5 min	[23]
lasB-R	GGTCCAGTAGTAGCGGTTGG			94°C – 30 s		
toxA-F	GGTAACCAGCTCAGCCACAT	352		55°C – 60 s		
toxA-R	TGATGTCCAGGTCATGCTTC			72°C – 90 s		
plcH-F	GAAGCCATGGGCTACTTCAA	307				
plcH-R	AGAGTGACGAGGAGCGGTAG					
exoS-F	CTTGAAGGGACTCGACAAGG	504				
exoS-R	TTCAGGTCCGCGTAGTGAAT					
aprA-F	ACCCTGTCCTATTCGTTCC	140	94°C – 5 min	30 cycles	72°C – 5 min	[24]
aprA-R	GATTGCAGCGACAACTTGG			94°C – 60 s		
				52°C – 60 s		
				72°C – 90 s		

PCR=Polymerase chain reaction

PCR products were visualized in 1.5% agarose gel by means of ultraviolet transilluminator. To confirm the specificity of the results, the amplified products of each gene were randomly selected, purified, submitted to sequencing, and further used as positive controls. For negative controls, water was added instead of DNA template.

## Results

### Prevalence of *P. aeruginosa* in canine clinical samples

A total of 24 *P. aeruginosa* isolates were found in positive samples from skin/soft tissue (6/39, 15.4%), ear canal (15/46, 32.6%), and urine (3/72, 4.2%). Pure cultures of *P. aeruginosa* were observed in all the urine samples. The majority of the samples from skin/soft tissue and ear canal showed pure culture of *P. aeruginosa*, while few samples showed a polymicrobial flora which was characterized by the strong dominance of *P. aeruginosa*.

### Antimicrobial profile

No resistance was found to CAZ, CN, ATM, and IMI, while low levels of resistance were found to ENR (n=1/24, 4.2%) and TZP (n=2/24, 8.3%). However, a high number of isolates showed intermediate susceptibility to ENR (n=10, 41.7% by disk diffusion method; n=11, 45.8% by gradient diffusion method) and IMI (n=7/24, 29.2%). A high degree of correlation was found between disk and gradient diffusion methods, and only two minor discrepancies relative to ENR and CN were recorded. In both cases, the gradient diffusion method showed an intermediate response while disk diffusion method revealed a susceptible profile (Table-2).

**Table 2:** Antimicrobial profiles of *Pseudomonas aeruginosa* by means of gradient diffusion method.

Antimicrobial	MIC breakpoint (μg mL^−1^)		R (%)	I (%)	MIC_50_ (μg mL^−1^)	MIC_90_ (μg mL^−1^)	% agreement with disk diffusion method	Number of isolates by error category		
	
	≤S	R≥						Very Major	Major	Minor
Enrofloxacin	0.5	4	1/24 (4.2)	11/24 (45.8)	0.5	2	95.8	0	0	1
Ceftazidime	8	32	0/24 (0)	2/24 (8.3)	2	8	100	0	0	0
Imipenem	2	8	0/24 (0)	7/24 (29.2)	2	4	100	0	0	0
Gentamicin	2	8	0/24 (0)	5/24 (20.8)	2	4	95.8	0	0	1
Aztreonam	8	32	0/24 (0)	1/24 (4.2)	4	8	100	0	0	0
Piperacillin-tazobactam	8/4	32/4	2/24 (8.3)	4/24 (16.7)	4	24	N.A.			

For each antibiotic, the number and the relative percentage of intermediate (I) or resistant (R) isolates were reported, in combination with MIC_50_ and MIC_90_ values. In the last two columns, the percentage of agreement with the disk diffusion method and the number of errors between the two methods were indicated.

### Phenotype screening of β-lactamase production

Five isolates were positive to AmpC β-lactamase test. Three of them (79-19, 50-20, and 52-20) were exclusively characterized by intermediate susceptibility to TZP, while two isolates (72-20 and 86-20) showed resistance to TZP and intermediate response to CAZ. Another isolate (60-20) showed an intermediate response against TZP, but it resulted negative to AmpC test; differently from the others, it was characterized by a reduced susceptibility to other b-lactams and also to ENR. No isolate was found positive for ESBLs and MBLs (Table-3).

**Table 3:** Phenotype screening tests for β-lactamases in *Pseudomonas aeruginosa.*

*Pseudomonas aeruginosa* isolates (internal nomenclature)	Response to b-lactams (I or R – MIC mg/mL)	Response to non b-lactams (I or R – MIC mg/mL)	Phenotype screening for β-lactamase		

			AmpC	ESBL	MBL
72-19	CAZ (I-12) TZP (R-64)	No	+	-	-
79-19	TZP (I-16)	No	+	-	-
82-19	IMI (I-4)	ENR (I-1)	-	-	-
50-20	TZP (I-24)	No	+	-	-
52-20	TZP (I-16)	No	+	-	-
60-20	ATM (I-16) IMI (I-4) TZP (I-24)	ENR (R->32)	-	-	-
65-20	IMI (I-6)	CN (I-4)	-	-	-
67-20	IMI (I-6)	No	-	-	-
80-20	IMI (I-4)	No	-	-	-
82-20	IMI (I-4)	CN (I-4) ENR (I-1)	-	-	-
84-20	IMI (I-4)	CN (I-4)	-	-	-
86-20	CAZ (I-16) TZP (R->256)	No	+	-	-

For each isolate, the intermediate (I) or resistance (R) response to β-lactams and non-β-lactams was indicated, including the relative MIC value. CAZ=Ceftazidima, TZP=Piperacillin-tazobactam, IMI=Imipenem, CN=Gentamicin, ENR=Enrofloxacin, ATM=Aztreonam

### Biofilm production

Five isolates (20.8%) were classified as strong biofilm producers, while 8 (33.3%) and 11 (45.8%) isolates were weak and intermediate biofilm producers, respectively. Among the strong producers, all ­isolates derived from the ear canal samples of the animals suffering otitis (Table-4).

**Table 4:** Distribution of *Pseudomonas aeruginosa* isolates based on their ability to produce biofilm and site of sampling.

Ability to produce biofilm	Number of isolates and relative percentage	Site of sampling		

		Urine	Ear canal	Skin/soft tissues
Weak	8/24 (33.3%)	0/3	7/15	1/6
Intermediate	11/24 (45.8%)	3/3	3/15	5/6
Strong	5/24 (20.8%)	0/3	5/15	0/6

### Molecular detection of pathogenic factors

All isolates (n=24, 100%) were positive for *lasB*, *aprA*, and *plcH* genes, while *exoS* and *toxA* were amplified in 21 (87.5%) and 22 (91.7%) isolates, respectively.

## Discussion

*P. aeruginosa* is a relevant opportunistic and difficult to treat pathogen due to its specific characteristics, such as the widespread environmental diffusion, the intrinsic resistance to many classes of antimicrobials, the high ability to acquire additional resistance mechanisms, and the wide range of virulence factors [25,26]. A low prevalence (4.2%) of *P*. *aeruginosa* was found in the urine of dogs suffering bladder infection, according to other authors which isolated *Pseudomonas* spp. in 2% [27], 1.3% [28], and 3.7% [29] of canine urinary tract infections. Instead, a discrete presence (32.6%) of *P. aeruginosa* was recorded in dogs with clinical signs of otitis, slightly higher if compared to the previous studies which reported a prevalence of 27% [30] and 23% [31]. Furthermore, in the skin and soft-tissue specimens, we found a higher prevalence of *P. aeruginosa* (15.4%) in comparison to the frequency isolation of 7.5% [30] and 10.6% [32] described by other studies.

The antimicrobial profiles of canine *P. aeruginosa* isolates are difficult to compare between different studies, due to many variables that represent a challenge for the harmonization of global data. In particular, antimicrobial methods used [33], interpretative criteria applied [34], body sites of sampling [35], geographical [36], and temporal differences [37] represent all variables that have to be taken into account. In the present study, the MICs values were determined by means of diffusion gradient method on agar, using a predefined antibiotic gradient strip. The previous studies on human clinical strains of *P*. *aeruginosa* showed that results obtained by gradient diffusion method correlated well with agar dilution [38], broth microdilution [39], and disk diffusion methods [40]. By comparing disk and gradient diffusion methods, no substantial discrepancies were observed in the present study, but only minor differences.

Specific veterinary breakpoints for *P. aeruginosa* were considered for CN, ENR, and TZP. Instead, human breakpoints were adopted for CAZ, IMI, and ATM, bearing well in mind that these antibiotics should be considered as last-resort options for human therapy (monobactams) or eventually recommended for a small number of infections (carbapenems, 3^rd^ generation cephalosporins) [41].

The widespread use of ENR in clinical practice of small animals has led to a continuous increase of resistant isolates of *P. aeruginosa* associated to dog infections [37,42]. In the past decade, high percentages of resistance to ENR were reported in Europe for *P. aeruginosa*, as well as described in Croatia (51%) [43], Greece (44%) [44], France (67%) [45], and Italy (43%) [46]. In the present study, only one isolate (4.2%) was found to be resistant to ENR, showing a MIC >32 mg/mL. This finding could be intended as a positive consequence of the national plans that promoted the responsible use of antibiotics in the past years. In France, the resistance to fluoroquinolones has shown a decreasing trend since 2013, due to the efforts to reduce the use of antibiotics in animals [45]. However, we could also interpret our data as a potential rising trend of the resistance of *P. aeruginosa* to ENR. Indeed, the high proportion of intermediate isolates (45.8% or 41.7% based on the method used) with MICs values (1-2 mg/mL) close to the susceptible category may be represented by bacterial variants with a low level of resistance that could accelerate the evolution toward high and clinically relevant resistance [47,48]. Although we have not investigated the mechanisms underlying the intermediate/resistance response to ENR, we could suppose the combined role of efflux pump overexpression and mutational variations of DNA gyrase/topoisomerase IV [49].

No isolate was resistant to CN in the present study, probably reflecting the minimal use of aminoglycosides in dogs due to their severe side effects. In this sense, low percentages (about 4%) of resistance to CN were detected worldwide in canine *P. aeruginosa* isolates [50-52], although some authors reported a higher presence of resistance, ranging from 26% [34] to 56% [53].

Among b-lactams antibiotics tested, a high percentage of isolates (29.2%) showed intermediate profile against IMI with MIC values ranging from 2 to 6 mg/mL. Carbapenem-resistant *P. aeruginosa* represents an emerging worldwide threat for human and animal health [54]. In the present study, none of these isolates were positive to the screening tests for β-lactamases and they were all susceptible to CAZ. The phenotype carbapenem-resistant/cephalosporin-susceptible represents an emerging profile in *P. aeruginosa* isolated from animal and human infection [55,56]. Therefore, resistance mechanisms different from the β-lactamase production have been investigated in the past years. In particular, the dysregulation of the efflux pumps system and the repression of the porin OprD expression represent two key mechanisms for the development of carbapenem-resistant *P. aeruginosa* in small animals [55]. However, carbapenemase-producing *P. aeruginosa* are characterized by higher MIC values in comparison with isolates lacking of this type of β-lactamase [34].

In the present study, 2 isolates (8.3%) showed resistance to TZP while 4 isolates showed intermediate susceptibility (16.7%), then revealing values comparable to what was reported by other authors [34,52]. As emerged by the screening tests for β-lactamases detection, AmpC was presumed to be the main mechanism responsible for the reduced susceptibility to TZP and also to CAZ in two isolates. The overexpression of AmpC β-lactamase in *P. aeruginosa* represents a common mechanism of resistance to the third-generation cephalosporins and to broad-spectrum penicillins [57], in association to other groups of β-lactamases, including ESBLs and MBLs that we have not detected by phenotype investigation.

Biofilm formation represents a well-investigated virulence factor of *P. aeruginosa*, promoting the persistence of the bacterial infection by the enhanced resistance to host immunity and antimicrobial drugs [58]. In the present study, most *P. aeruginosa* isolates resulted in being weak or intermediate biofilm producers, while the strong biofilm producers were derived exclusively from the ear canal samples. In this sense, biofilm production represents a common feature of *P. aeruginosa* involved in dog otitis, then facilitating the persistence of infection [59,60].

The extracellular pathogenic factors play a key role in *P. aeruginosa* infection and the most investigated are represented by exotoxin A, phospholipase C, elastase, alkaline protease, and type III secretion system [61]. Few data are available in veterinary literature about the occurrence of these factors in *P. aeruginosa* clinical isolates from companion [62] and farm animals [63-65]. In the present study, a high occurrence was found for all the pathogenic markers that we investigated, suggesting the elevated virulence potential of the isolates, as well as reported in human clinical isolates [66-68].

All the factors we investigated have a key role in the invasion, colonization, and establishment of infection by *P. aeruginosa*. Elastase and alkaline protease are characterized by proteolytic activity against host extracellular matrix proteins [69]. Phospholipase C and exotoxin A exert the cytotoxic activity by hydrolysis of cell membrane lipids [70] and inhibition of cell protein synthesis [71], respectively. ExoS is a bifunctional enzyme of the type-III protein complex and it is involved in cytotoxic activity and in the ­stimulation of actin reorganization of cells [72]. In addition, these pathogenic elements favor the dissemination of *P. aeruginosa* through various mechanisms that negatively interfere with host immunity [73-77].

## Conclusion

In the present study, the antimicrobial profiles of *P. aeruginosa* isolates from canine clinical samples were not characterized by an alarming resistance. However, the high presence of isolates with intermediate susceptibility to ENR could represent an early warning signal to monitor over time. Furthermore, the finding of reduced susceptibility to antibiotics not authorized for veterinary use, such as carbapenems, was indicative of the importance of the One Health approach to understand and to contrast the emerging diffusion of resistant pathogens. Moreover, the presence of isolates with strong ability to produce biofilm must be taken into account in the interpretation of the antimicrobial susceptibility profile. Besides, the high prevalence of the extracellular pathogenic factors was indicative of the potential virulence of the isolates.

## Authors’ Contributions

JH: Laboratory work, data analysis, and writing of the manuscript. FM: Planning of the study, laboratory work, data analysis, and writing of the manuscript. CED: Design, analysis, and interpretation of the molecular biology data and revision of the manuscript. GA: Sample collection and critical revision of the manuscript. GM: Critical revision of the manuscript. PG: Sample collection. PGT: Data analysis and critical revision of the manuscript. All authors read and approved the final manuscript.
